# Enhancing the patient involvement in outcomes: a study protocol of personalised outcome measurement in the treatment of substance misuse

**DOI:** 10.1186/1471-244X-13-337

**Published:** 2013-12-16

**Authors:** Paula CG Alves, Célia MD Sales, Mark Ashworth

**Affiliations:** 1 Instituto Universitário de Lisboa (ISCTE-IUL), Cis-IUL, Sala 2w17, Avª Forças Armadas, Edifício ISCTE, Lisboa 1649-026, Portugal; 2 Division of Health and Social Care Research, School of Medicine, King’s College London, 7th Floor Capital House, 42 Weston Street, London SE1 3QD, UK; 3 Departamento de Psicologia, Universidade de Evora (CIEP-UE), Colégio Pedro da Fonseca, R. Barba Rala, 1, PITE, Évora 7005-345, Portugal

**Keywords:** Patient involvement, Personalised outcome measurement, Patient perspective, Patient-generated outcome measures, Individualized outcome measures, Substance misuse treatment

## Abstract

**Background:**

Involving patients in treatment is becoming increasingly popular in mental health [Sales & Alves: Personalized evaluation of psychological treatments: A review of tools and research designs, submitted]. However, in substance misuse treatment settings, the patient perspective about treatment tends to be overlooked. This has been cited as a key priority by Orford et al. [Addiction, 103: 875-885, 2008] who included patient feedback about treatment as one of ten areas requiring an urgent paradigm shift in addiction research and practice.

This project will apply an innovative method to involve substance misuse patients in psychological therapies, by asking them to suggest topics to evaluate their treatment. These topics suggested by patients can be written as a list of personalised items, so-called as patient-generated outcome measures (PGOM). Despite its patient-friendly features, PGOM’s have never been used in this population, which is what this project aims to overcome.

**Methods/design:**

This project is part of an International Exchange Platform on Personalising Addiction Treatment. Data will be collected in two phases (pre-post study and focus groups with patients) to explore the following:

1). How reliable and sensitive to change are PGOM’s and standardised measures in substance misuse treatment?

2). Do PGOM’s add relevant information to standardised measures?

3). What are the views of substance misuse patients about personalised outcome assessment?

4). Development of guidelines on using PGOM’s in this population

**Discussion:**

This research will potentially demonstrate the diversity of personal problems among patients seeking substance misuse treatment, suggesting the relevance of PGOM as a method to personalise outcome measurement and, ultimately, guiding treatment provision. It is expected that, as in previous studies, PGOM’s will be perceived as helpful and patient-friendly tools, where patients may express their own concerns in a semi-structured setting. Similarly to other populations, we also expect PGOM’s to be reliable, valid and sensitive to clinical changes in substance misuse treatment, as well as more content informative than their standardised counterparts. If these results are achieved, we might hypothesize that PGOM’s are a potentially valid supplement to traditional standardised scales, by providing a closer insight to what motivates patients to participate in substance misuse treatment programmes.

## Background

Involvement of the patient in mental health care is currently a subject of intense debate [Sales & Alves: Personalized evaluation of psychological treatments: A review of tools and research designs, submitted]. This approach involves listening to the patient perspective, taking into account his particular needs and his views about health care processes [1,2]. Patient involvement with treatment is already being implemented in various mental health settings, such as psychiatric units [3] or primary mental health care [4] with encouraging results. In substance misuse treatment settings, the involvement of patients is also deemed to be of great importance, both by researchers [5] and policy makers (e.g. Institute on Drugs and Drug Addiction, Portugal). However, in this context, the patient perspective still remains often overlooked and our project will contribute to reducing this gap.

Patients can be directly involved in healthcare at different levels, such as service management, community initiatives, public policy and treatment provision/planning. When it comes to being involved with treatment, there are various strategies that can be applied. On the one hand, the patient perspective may be accessed with traditional standardised tools that encompass pre-set and psychometrically determined items. This is the most commonly used approach. Alternatively, we might ask patients to propose the content of the items, thus building a personalised questionnaire, containing his or her personal problems or goals for therapy. Ashworth and collaborators defined these as “patient-generated outcome measures” (PGOM), put other way, “questionnaires where the items to be measured are defined by the patient” [6].

Due to their open-ended structure, PGOM’s encourage patients to express their point of view, hence allowing a “personalised evaluation” of patient’s condition. For such reason, we designate as “personalised outcome assessment” all types of outcome assessment that use PGOM’s, either alone or as an increment to traditional standardised measures [3].

The use of personalised outcome assessment has been growing in popularity in the last decades [Sales & Alves: Personalized evaluation of psychological treatments: A review of tools and research designs, submitted]. For instance, previous studies have shown that psychotherapists find the routine use of PGOM’s useful for treatment planning and decision-making [3,7]. Psychometrically, PGOM’s appear to be more sensitive to detect clinical change when compared with standardised scales [8]. Also in primary care, a recent study found that PGOM’s were useful to identify different sub-types of patients with a physical condition, based on their patient-generated, self-reported problems [9]. From a policy point of view, international health authorities, such as the American Psychological Association (http://www.divisionofpsychotherapy.org/continuing-education/task-force-on-evidence-based-therapy-relationships/conclusions-of-the-task-force/) or the United Kingdom’s National Institute for Health and Care Excellence (http://publications.nice.org.uk/quality-standard-for-service-user-experience-in-adult-mental-health-qs14/quality-statement-5-using-views-of-service-users-to-monitor-and-improve-services) also advocate for the active involvement of patients in their own treatment (the ‘expert patient’) as a best-practice principle.

Despite this overall interest in patient involvement, the patient perspective in substance misuse treatment settings remains relatively unexplored and unheard (Orford, [5]). According to Orford [1], this is one of the ten aspects needing an urgent shift in the provision of substance treatment, where patients tend to be misinformed about the rationale behind their treatment plans [10]. Hence, listening to patients is a powerful strategy to explore what patients specifically need from treatment services, potentially avoiding outcomes or early treatment drop-out. As a consequence, it is extremely vital to study methodologies to overcome this limitation.

To our knowledge, PGOM’s have never been used in substance misuse treatment settings and it is unknown whether these personalised measures are useful to this population. Having this in mind, and acknowledging the importance of this topic, in this project we will explore to what extent a personalised outcome measurement system, with PGOM’s, can be used in substance misuse.

To achieve this goal, we will, on the one hand, investigate if PGOM’s are psychometrically robust to measure psychological changes in this context; and, on the other hand, to explore the experiences of patients with those measures. Guidelines for future use and recommendations for clinical policies will be derived from these data in an International Platform for Personalising Addiction Treatment.

More specifically, our aims are:

1. To study the quantitative and qualitative properties of PGOM’s in this population

2. To investigate the experiences of patients using personalised outcome measurement and the feasibility of this approach, from their point of view (e.g. easiness of understanding, comfort in use)

3. To exchange knowledge about personalised outcome measurement towards the development of a guidance document to use this approach in substance misuse treatment settings (e.g. when to use PGOM’s and under what circumstances, recommended frequency of use)

## Methods/design

To achieve the aforementioned aims, we will collect data at four units specialising in substance misuse treatment in Portugal, of which two target misuse of drugs (licit/illicit) and two misuse of alcohol. Three of these are outpatient units and one is a therapeutic community. All these units provide assessment, treatment and advice for addiction-related problems.

### Sample and inclusion/exclusion criteria

The target sample for this project includes male and female adult (>18 years old) patients seeking treatment for drug and alcohol dependence at the four treatment sites. All patients starting (first treatment episode) or being re-admitted (new treatment episode after treatment drop-out) for treatment at these sites will be invited to participate in the study. Re-admission for treatment occurs whenever patients have dropped-out of the last treatment episode for a period longer than 8–12 months. Patients are excluded based on 1) lack of interest or motivation to take part and 2) being non-Portuguese speakers.

On sample size, a minimum set of 100 pairs of questionnaires (pre-post) is expected to provide tight confidence intervals on correlations between the measures [8].

### Measurements

Four outcome measures of psychological well-being will be used, of which two are PGOM’s and the other two are standardised. The PGOM’s are PSYCHLOPS [6], a self-report questionnaire for patients to generate and score two items about Problems, one about Function and one about Well-being; and PQ [Elliott: Simplified Personal Questionnaire Procedure, unpublished] [11], an interview-based questionnaire for patients to generate, rank order and score an unlimited list of personal complaints, in whichever topic. Two PGOM’s were selected to explore patients’ preferences about PGOM’s with different characteristics (e.g. pre-set vs. non pre-set domains).

The standardised measures are CORE-OM [12,13], a 34-item self-report measure of psychological well-being. CORE-OM was preferred over other measures used in this population because it is mental health specific (unlike SF Health Survey) and covers risk behaviours (unlike SF Health Survey & BSI); and PHQ-9 [14], a self-report mood disorders scale often included with CORE-OM in outcome studies. A fifth outcome instrument, TOP [15], will be included to collect data about the patient’s drug-related situation. TOP is a brief scale to monitor and evaluate key domains for drug-treatment, namely: Drug and alcohol use; Injecting risk behaviours; Offending and criminal involvement; and Health and Social functioning.

PQ, CORE-OM and PHQ-9 will be input in IPPS, the software developed by our team [3,4] to facilitate data management.

### Data collection strategy

Quantitative and qualitative data will be collected for this project in two phases. The first involves a pre-post study that will follow a methodology similar to previous outcome studies in the field of substance misuse [16]. It will comprise 2 evaluation moments:

• Pre-treatment (evaluation 1) administration of PQ, PSYCHLOPS, CORE-OM and PHQ-9, presented in randomised order, as well as TOP. This pre-treatment moment will thus involve the generation of the items in PGOM’s, followed by their scoring; the scoring of the standardised measures; and completion of a socio-demographic and treatment history form.

• Three to five months after treatment entry (evaluation 2), in which the aforementioned outcome measures will be re-administered to the same patients who completed evaluation 1 (paired sample). Regarding PGOM’s, in this second evaluation moment, patients may chose to delete and/or add new items.

**Figure 1:**
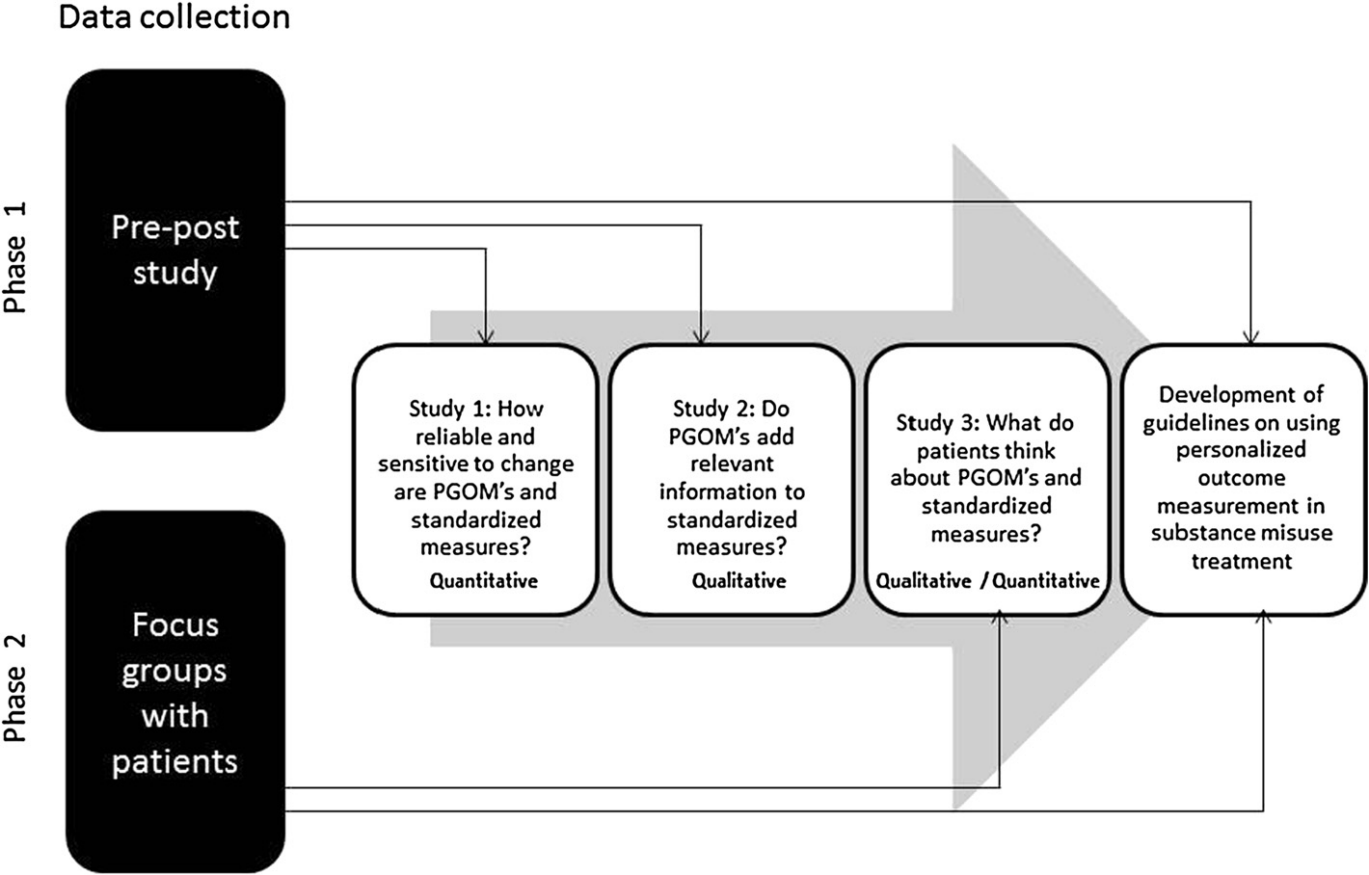
Study flow and research questions to be explored during the project.

The second phase of data collection will comprise focus groups with patients that participated in the pre-post study. The data collected in these two phases will be used in 3 studies, as explained in the next sections. See Figure 1 for a diagram with the study flow.

### Study 1: How reliable and sensitive to change are PGOM’s and standardised measures in substance misuse treatment?

In this quantitative study, the pre-post scores provided by patients in PGOM’s (PQ and PSYCHLOPS) and standardised (CORE-OM and PHQ-9) measures will be compared according to several psychometric parameters of reliability and change sensitivity, namely: Internal reliability; Mean improvement rates; Sensitivity to change (effect size); and Convergent validity. This analytical procedure will replicate the methodology adopted by Ashworth et al. in a similar study [8].

### Study 2: Do PGOM’s add relevant information to standardised measures?

In this study, we will identify the range and nature of problems generated by PGOM’s and compare these with domains covered by standardised measures. This study will involve the categorization of patient-generated items provided in PQ & PSYCHLOPS at both evaluation moments. The categorization will follow a grounded theory approach and will be performed independently by two judges (post-graduate students in addiction psychology). Disagreements will be discussed until a final list of categories of problems is obtained. Then, as in Ashworth et al. [5], we will explore the content similarity between the categories elicited by PQ & PSYCHLOPS vs. domains covered by standardised measures.

### Study 3: What are the views of substance misuse patients about personalised outcome assessment?

Focus groups will be implemented to study what patients think about PGOM. As in Crawford et al. [17], patients recruited for the pre-post study will be invited and evenly distributed into four focus groups (i.e. at least one group per research site). We expect a minimum of 5 patients per each focus group. At these groups, we will review the 5 outcome measures used in the pre-post study and discuss their experiences whilst answering to them, based on questions proposed by Stone & Elliott [18]: the helpful, hindering and difficult but OK aspects of the assessments, including suggestions for improvement. Content analysis will be conducted on the topics elicited. Additionally, patients will be also asked to rate each measure (in a 5-point scale) for: Relevance of covered topics; Comfort with information disclosed; Applicability in this population; Ease of understanding; and Overall satisfaction with use.

### Development of guidelines

The final stage of this project involves the development of guidelines on personalised outcome measurement, to inform therapists on how to involve patients in substance misuse treatment, as proposed by health authorities [19]. These guidelines will be developed in the context of a research network, the International Exchange Platform on Personalising Addiction Treatment and will be based on the results of this project and previous / on-going work of the research network members. It will focus on: The patient perspective about personalised outcome measurement; Potentialities and practical aspects of personalised outcome measurement (e.g. which measures; when to evaluate); Best-practice principles, challenges and barriers to its implementation; and Future research recommendations.

### Ethics and data protection

Ethical approval for this project has been obtained from the ethics committee of the Health Region Administration of Lisbon (ARSLVT), Portugal. All patient data will be de-identified and the patient’s identity and personal details will be omitted whenever required (e.g. as patients are asked to generate their own contents, they may provide, written or verbally, details that identify others, such as names or places^1^).

## Discussion

This project was designed using an innovative approach to involving patients in the evaluation of (their own) treatment, the personalised outcome measurement approach. This approach provides patients with the opportunity to put some input in which topics are used to evaluate and monitor the progress of their treatment.

As previously explained, the personalised outcome measurement strategy has never been implemented in substance misuse treatment settings. For this reason, we believe that this approach is a potential tool to help shifting the paradigm towards a greater involvement of patients in substance misuse treatment, potentially leading to a better adjustment of treatment to the patient’s needs and, ultimately, avoiding early treatment drop-out.

This study will also provide data to evaluate the reliability, validity and sensitivity to change of PGOM’s in comparison with traditional measures of psychological well-being, in this population. In previous studies with other clinical populations, the results showed that PGOM’s are potentially powerful tools for outcome measurement purposes in therapeutic practice, as well as group and case study research [Elliott, Wagner, Sales, Rodgers, Alves & Café: Psychometrics of the Personal Questionnaire: A Client-Generated Outcome Measure, in preparation]. Our findings will contribute to this growing body of research, complementing this important work.

Besides studying the quantitative and qualitative properties of PGOM’s, this project takes a step further by asking patients about their experience whilst responding to different types of outcome measures. This is an important part of the project, for it will show which measures are preferred by patients and what are their perspectives on implementing this outcome measurement approach in their treatment.

By studying the psychometric properties of PGOM’s in this population and exploring what patients think and feel about using them, we will be able to generate, in an international and multidisciplinary platform, a guidance document that will help clinicians and researchers taking this personalised strategy on board. The development of clinical guidelines is a procedure often derived by experts (either clinicians or researchers) based on the evidence available, overlooking the patient perspectives. In our project, however, we will overcome this limitation by combining, on the one hand, the findings of our empirical studies, but also the perspectives of patients about each measure.

Data collection for this project began in April 2013 and we anticipate it to end in mid 2014. Analyses are expected to begin in the second half of 2014. To date, approximately 40% of the target sample has already completed the pre-treatment evaluation moment. The re-administration of the instruments (post-treatment evaluation) has begun as well and the first focus groups are expected to be held between December 2013 and January 2014.

It is also worth mentioning that, so far, only 4 out of 40 patients invited to take part in the study declined to participate, which shows the high level of adherence and acceptance that this project is having in this setting.

To date, our experience in this project has shown us that both service providers and patients are motivated to contribute to reducing the gap between science and practice, by joining efforts to improve the treatment which is provided in real clinical settings. Ultimately, we believe that treatment provision, and the evaluation of its outcomes, should take on board the perspectives of all parties involved in the process, from service managers, therapists and, most importantly, patients, so that healthcare may be designed to meet the real needs of its target population.

## Endnote

^1^Examples: “I think that my neighbor John Stuart hates me”, “I don’t trust in Dr. Bolton anymore”.

## Competing interests

The authors declare that they have no competing interests in the preparation of this study protocol or the presentation of information.

## Authors’ contributions

The idea for this study was conceived and designed by PCGA, as part of her PhD project, and CMDS and MA, as the academic supervisors of the project. PCGA drafted the manuscript and is currently managing data collection and the implementation of the study in general. CMDS and MA also contributed for the draft of the manuscript. All authors read and approved the final manuscript.

## Pre-publication history

The pre-publication history for this paper can be accessed here:

http://www.biomedcentral.com/1471-244X/13/337/prepub
